# 144. Clinical Validation and Performance of a T-cell Immunosequencing Assay to Identify Past SARS-CoV-2 Infection

**DOI:** 10.1093/ofid/ofab466.144

**Published:** 2021-12-04

**Authors:** Sudeb C Dalai, Jennifer N Dines, Thomas M Snyder, Rachel M Gittelman, Tera Eerkes, Pashmi Vaney, Sally Howard, Kipp Akers, Lynell Skewis, Anthony Monteforte, Pamela R Witte, Cristina Wolf, Hans Nesse, Jia Qadeer, Sarah Duffy, Emily Svejnoha, Caroline Taromino, Michael Kagen, Ian M Kaplan, John Alsobrook, Thomas Manley, Lance Baldo

**Affiliations:** 1 Adaptive Biotechnologies and Stanford University School of Medicine, Seattle, Washington; 2 Adaptive Biotechnologies, Seattle, Washington; 3 Kagen MD, Park City, Utah

## Abstract

**Background:**

Our understanding of the SARS-CoV-2 immune response has critical gaps that are inadequately addressed with available tools. We report the clinical performance of T-Detect COVID, the first T-cell assay to identify prior SARS-CoV-2 infection using T-cell receptor (TCR) sequencing and repertoire profiling from whole blood samples.

**Methods:**

The T-Detect COVID assay combines high-throughput immunosequencing of the TCRß gene from blood samples with a statistical classifier demonstrating 99.8% specificity for identifying prior SARS-CoV-2 infection. The assay was employed in several retrospective and prospective cohorts to assess primary and secondary Positive Percent Agreement (PPA) with SARS-CoV-2 RT-PCR (N=205; N=77); primary and secondary Negative Percent Agreement (NPA; N=87; N=79); PPA compared to SARS-CoV-2 serology (N=55); and pathogen cross-reactivity (N=38). The real-world performance of the test was also evaluated in a retrospective review of test ordering (N=69) at a single primary care clinic in Park City, Utah.

**Results:**

In validation studies, T-Detect COVID demonstrated high PPA (97.1% ≥15 days from diagnosis) in subjects with prior PCR-confirmed SARS-CoV-2 infection; high NPA (~100%) in SARS-CoV-2 negative cases; equivalent or higher PPA with RT-PCR compared to two commercial EUA antibody tests; and no evidence of pathogen cross-reactivity. Review of assay use in a single clinic showed 100% PPA with RT-PCR in individuals with past confirmed SARS-CoV-2 vs. 85.7% for antibody testing, 100% agreement with positive antibody results, and positive results in 2/4 convalescent subjects with seroreversion to a negative antibody. In addition, 12/69 (17.3%) individuals with absent or negative RT-PCR tested positive by T-Detect COVID, nearly all of whom had compatible symptoms and/or exposure. TCR positivity was observed up to 12+ months (median 118 days) from the date of positive RT-PCR.

**Conclusion:**

A T-cell immunosequencing assay shows high clinical performance for identifying past SARS-CoV-2 infection from whole blood samples. This assay can provide additional insights on the SARS-CoV-2 immune response, with practical implications for clinical management, risk stratification, surveillance, assessing vaccine immunity, and understanding long-term sequelae.

**Disclosures:**

**Sudeb C. Dalai, MD, PhD**, **Adaptive Biotechnologies** (Employee, Shareholder) **Jennifer N. Dines, MD**, **Adaptive Biotechnologies** (Employee, Shareholder) **Thomas M. Snyder, PhD**, **Adaptive Biotechnologies** (Employee, Shareholder) **Rachel M. Gittelman, PhD**, **Adaptive Biotechnologies** (Employee, Shareholder) **Tera Eerkes, PhD**, **Adaptive Biotechnologies** (Employee, Shareholder) **Pashmi Vaney, PhD**, **Adaptive Biotechnologies** (Employee, Shareholder) **Sally Howard, PhD**, **Adaptive Biotechnologies** (Employee, Shareholder) **Kipp Akers, PhD**, **Adaptive Biotechnologies** (Employee, Shareholder) **Lynell Skewis, PhD**, **Adaptive Biotechnologies** (Employee, Shareholder) **Anthony Monteforte, PhD**, **Adaptive Biotechnologies** (Employee, Shareholder) **Pamela R. Witte, PhD**, **Adaptive Biotechnologies** (Employee, Shareholder) **Cristina Wolf, PhD**, **Adaptive Biotechnologies** (Employee, Shareholder) **Hans Nesse, PhD**, **Adaptive Biotechnologies** (Employee, Shareholder) **Jia Qadeer, PhD**, **Adaptive Biotechnologies** (Employee, Shareholder) **Sarah Duffy, PhD**, **Adaptive Biotechnologies** (Employee, Shareholder) **Emily Svejnoha, PhD**, **Adaptive Biotechnologies** (Employee, Shareholder) **Caroline Taromino, PhD**, **Adaptive Biotechnologies** (Employee, Shareholder) **Ian M. Kaplan, PhD**, **Adaptive Biotechnologies** (Employee, Shareholder) **John Alsobrook, MD**, **Adaptive Biotechnologies** (Employee, Shareholder) **Thomas Manley, MD**, **Adaptive Biotechnologies** (Employee, Shareholder) **Lance Baldo, MD**, **Adaptive Biotechnologies** (Employee, Shareholder, Leadership Interest)

